# Author Correction: Prediction of oxygen requirement in patients with COVID-19 using a pre-trained chest radiograph xAI model: efficient development of auditable risk prediction models via a fine-tuning approach

**DOI:** 10.1038/s41598-023-31333-0

**Published:** 2023-03-15

**Authors:** Joowon Chung, Doyun Kim, Jongmun Choi, Sehyo Yune, Kyoung Doo Song, Seonkyoung Kim, Michelle Chua, Marc D. Succi, John Conklin, Maria G. Figueiro Longo, Jeanne B. Ackman, Milena Petranovic, Michael H. Lev, Synho Do

**Affiliations:** 1 Department of Radiology, Massachusetts General Brigham and Harvard Medical School, Boston, MA USA; 2 Department of Radiology, Samsung Medical Center, Sungkyunkwan University School of Medicine, 81 Irwon-Ro, Gangnam-Gu, Seoul, 06351 Republic of Korea

Correction to: *Scientific Reports* 10.1038/s41598-022-24721-5, published online 07 December 2022

The original version of this Article contained an error in the spelling of the author Kyoung Doo Song which was incorrectly given as Kyungdoo Song.

Additionally, an Affiliation was omitted for Kyoung Doo Song.

The correct affiliations are listed below.

Department of Radiology, Massachusetts General Brigham and Harvard Medical School, Boston, MA, USA.

Department of Radiology, Samsung Medical Center, Sungkyunkwan University School of Medicine, 81 Irwon-Ro, Gangnam-Gu, Seoul, 06351, Republic of Korea.

The original Article has been corrected.

